# Effects of rehabilitation interventions for old adults with long COVID: A systematic review and meta-analysis of randomised controlled trials

**DOI:** 10.7189/jogh.14.05025

**Published:** 2024-09-06

**Authors:** Jie Deng, Chenyuan Qin, Minjung Lee, Yubin Lee, Myoungsoon You, Jue Liu

**Affiliations:** 1 Department of Epidemiology and Biostatistics, School of Public Health, Peking University, Beijing, China; 2 Dental Research Institute, School of Dentistry, Seoul National University, Seoul, Republic of Korea; 3 Department of Public Health Sciences, Graduate School of Public Health, Seoul National University, Seoul, Republic of Korea; 4 Institute of Health and Environment, Seoul National University, Seoul, Republic of Korea; 5 Institute for Global Health and Development, Peking University, Beijing, China

## Abstract

**Background:**

There is limited evidence on the effectiveness of the existing rehabilitation interventions for old adults with long coronavirus disease (COVID), which is of particular concern among old adults.

**Methods:**

We systematically searched studies published in PubMed, EMBASE, Web of Science, Scopus, and Cochrane Library databases from their inception to 15 November 2023. Randomised controlled trials (RCTs) compared rehabilitation interventions with other controls in old adults (mean/median age of 60 or older) with long COVID were included. We performed a meta-analysis to compare the effects of the rehabilitation interventions with the common control group. Mean difference (MD) or standardised mean difference (SMD) with its 95% confidence intervals (CI) were used as summary statistics. Moreover, subgroup analyses based on the intervention programmes, the severity of acute infection, and the age of participants were carried out.

**Results:**

A total of 11 RCTs involving 832 participants (64.37 ± 7.94 years, 52.2% were men) were included in the analysis. Compared with the control groups, rehabilitation interventions significantly improved 6-minute walking test (6 MWT; MD = 15.77 metres (m), 95% CI = 5.40, 26.13, *P* < 0.01), 30-second sit-to-stand test (MD = 4.11 number of stands (n), 95% CI = 2.46, 5.76, *P* < 0.001), all aspects of quality of life, independence in activities of daily living (SMD = 0.31, 95% CI = 0.14, 0.48, *P* < 0.001), and relieved fatigue (SMD = −0.66, 95% CI = −1.13, −0.19, *P* < 0.01), depression (SMD = −0.89, 95% CI = −1.76, −0.02, *P* < 0.05) and anxiety (SMD = −0.81, 95% CI = −1.58, −0.05, *P* < 0.05). However, the improvement of hand grip strength and pulmonary function was not statistically significant (*P* > 0.05). Subgroup analyses showed that improvements in 6 MWT, fatigue, anxiety, and depression were more pronounced in old patients who received exercise training, while those who received respiratory rehabilitation had more pronounced improvements in pulmonary function and quality of life.

**Conclusions:**

Old adults with long COVID who underwent rehabilitation interventions experienced significant improvement in functional capacity, fatigue, quality of life, independence in activities of daily living, and mental health outcomes compared with usual/standard care. These findings suggest that screening, management, and rehabilitation interventions for long COVID in older adults should be strengthened to improve their complete health status and functional status, thereby reducing the long-term disease burden caused by long COVID and fostering healthy aging during the post-pandemic era.

The pandemic of coronavirus disease 2019 (COVID-19) has posed a significant impact on global public health, particularly old adults who were more vulnerable to severe illness and complications following COVID-19 infection [1]. Although survivors recovered from the acute phase of COVID-19, many of them were facing various ongoing symptoms, which were defined as post-COVID-19 condition (PCC) or long COVID, impacted functioning and activities of daily living, encompassing physical, psychological, and cognitive functions [2,3]. A scoping review of 50 studies suggested that long COVID was a heterogeneous condition, with a spectrum of over 100 reported symptoms, the incidence of which ranged from 10 to 80% [4]. Long COVID has emerged as an important public health issue that could increase the existing burden of diseases and health care resources globally [5].

Long COVID is of particular concern among older adults. Previous studies showed that older patients faced an increased risk of morbidity and mortality due to COVID-19, and were at a higher risk of long COVID compared to younger patients [1,6]. Besides, COVID-19 might worsen or trigger chronic conditions commonly found in older people, such as cardiovascular diseases, respiratory diseases, neurodegenerative conditions, and functional decline [1]. Furthermore, lockdowns and other restrictions, as well as the possibility of losing a spouse or loved one during the pandemic, may also contribute to the mental and physical decline of older persons [1]. Aging present is a growing problem globally. According to the World Health Organization (WHO), the number of people aged 60 years and older was one billion in 2019 and will increase to 1.4 billion by 2030 [7]. The older population bears an additional burden in the face of long COVID, and the size of older people affected by long COVID is enormous if estimated by the prevalence rate of 10–80%, which could pose a great burden to the health care system [4]. Thus, a critical consideration is necessary for the management and rehabilitation of long COVID in the aged population, which would also help foster healthy aging during the pandemic towards a Decade of Healthy Ageing (2021–2030) [8].

Effective rehabilitation interventions for long COVID are critical, especially in the elderly, a high-risk population. The conclusions of existing studies on rehabilitation interventions for long COVID were inconsistent. According to previous reviews, rehabilitation interventions for patients with long COVID encompassed a range of approaches such as respiratory exercises, aerobic training, strength exercises, psychological support, medicine treatment, etc. [9–11]. The latest systematic review with meta-analysis (1244 participants of 14 trials; median (interquartile range (IQR)) age, 50 (47–56) years) suggested that rehabilitation interventions were associated with improvements in functional exercise capacity, dyspnoea, and quality of life, with a high probability of improvement compared with the current standard care [9]. However, most of the existing studies assessing the effectiveness of rehabilitation interventions for long COVID were targeted at the general population, and very limited high-quality evidence has specifically addressed the rehabilitation interventions for old adults recovering from COVID-19. McCarthy et al. conducted a systematic review and meta-analysis (570 older adults of 12 studies) and the results showed that multidisciplinary rehabilitation may result in improved functional outcomes on discharge from rehabilitation units/centres for older adults with COVID-19, but the limitation is that no trial studies were included in this review and none of the included studies followed up patients after discharge or reported on long term effects of COVID-19 on discharge from rehabilitation units [10]. Therefore, enhancing research to provide high-quality evidence on rehabilitation interventions for older adults with long COVID is essential.

Rehabilitation interventions for old adults played a crucial role in addressing challenges posed by long COVID. The general aim of geriatric rehabilitation is to improve the complete health status and functional status of older patients and to prevent and treat the physical, functional, and psychological impairments resulting from COVID-19 [12]. Therefore, these questions should be considered and addressed: what are the existing rehabilitation interventions for old adults with long COVID? Compared to standard care, some rehabilitation interventions have shown symptomatic and functional improvements in the general population with long COVID, however, what are the effects of these existing interventions for old adults, a specific and vulnerable group? There is a paucity of evidence on these questions. Therefore, we conducted a systematic review and meta-analysis of the existing randomised controlled trials (RCTs), aimed to comprehensively summarise the pattern and effectiveness of current rehabilitation interventions for old adults with long COVID.

## METHODS

### Search strategy

We searched studies published in PubMed, EMBASE, Web of Science, Scopus and Cochrane Library databases from their inception to 15 November 2023 without language restrictions. We used a search strategy with a combination of Medical Subject Headings (MeSH) terms and key terms (words in the title, keywords or abstract of the manuscript) to identify potential studies. Examples of the keywords we used were the following terms: (‘long covid’ OR ‘post covid’ OR ‘long-covid’ OR ‘post-covid’ OR ((‘long-term’ OR ‘post-acute’ OR ‘sequela’ OR ‘sequala’) AND (‘SARS-CoV-2’ OR ‘COVID-19’))) AND (‘rehabilitation’ OR ‘recovery’ OR ‘management’ OR ‘telehealth’ OR ‘exercise’ OR ‘training’ OR ‘therapy’ OR ‘medicine’ OR ‘physical’) AND (‘randomized controlled trial’ OR ‘clinical trial’ OR ‘intervention’ OR ‘RCT’). The detailed search strategy is shown in Appendix 1 in the **Online Supplementary Document**. Furthermore, we manually examined the reference lists of the studies included to identify any additional studies not found during the electronic search. We used EndNoteX9.3.3 (Thomson Research Soft, Stanford, USA) to manage records.

This study was strictly performed according to the Preferred Reporting Items for Systematic Reviews and Meta-Analyses (PRISMA) which is shown in Appendix 2 in the **Online Supplementary Document**. [13]. The protocol of this study has been registered in the PROSPERO database (CRD42023412605).

### Inclusion and exclusion criteria

We included RCTs that compared rehabilitation interventions such as respiratory rehabilitation, aerobic training, exercise training, and other rehabilitation programmes with either placebo, usual care, or control in old adults (with a mean or median age of 60 or older) with long COVID. No limitations were imposed regarding the presence of comorbidities or concurrent medication use during the rehabilitation programmes. The following studies were excluded: (1) irrelevant to the subject of the systematic review and meta-analysis; (2) insufficient data that cannot be addressed after contacting the authors; (3) duplicate studies; (4) non-RCT studies, qualitative researches, reviews, editorials, conference papers, case reports or animal experiments; or (5) the age of participants less than 60 years. Studies were identified by two investigators independently following the criteria above, while discrepancies were solved by consensus or with a senior research team member.

### Data extraction

The following data was extracted from the eligible studies: (1) basic information about the studies, including the publication year, authors, study conducting time, and country; (2) characteristics of participants (sample size, age, sex ratio, comorbidity, severity in acute infection et al.), intervention programmes, control, and outcomes. Data extraction was conducted by two investigators independently. As for missing or unclear data, try to solve it by consulting with panel members or contacting the corresponding authors to gain the original data.

### Outcomes

The primary outcome was functional capacity, mainly measured with the 6-minute walking test (6MWT, metre (m)). Secondary outcomes included functional capacity (assessed by 30-second sit-to-stand test (30 seconds STS, number of stands(n) and hand grip strength (HGS, kg)), pulmonary function (measured by the forced expiratory volume in one second/forced vital capacity (FEV1/FVC, %)), fatigue, quality of life, independence in activities of daily living, anxiety, and depression. Fatigue was assessed by the Fatigue Severity Scale (FSS), which consisted of nine questions that measured the patient’s perception of how fatigue affects their daily activities, with the higher score, the more fatigue [14]. Quality of life was assessed through the Short Form 36 Health Survey Questionnaire (SF-36), which consisted of eight domains, including physical functioning, role-physical, bodily pain, general health, vitality, social functioning, role-emotional, and mental health [15]. Scores for each domain ranged from 0 to 100, and higher scores indicated better quality of life. Independence in activities of daily living was assessed through the Functional Independence Measure (FIM), Katz Index of Independence in Tasks of Everyday Living (KATZ), and Barthel Index (BI). Depression was assessed by the Hamilton Anxiety and Depression Scale (HADS) and Self-rating Depression Scale (SDS) [16,17]. Anxiety was assessed by the HADS and Self-rating Anxiety Scale (SAS) [16,18]. All of the primary and secondary outcomes were assessed at the earliest available time point after the completion of the rehabilitation programme.

### Quality assessment

Two independent investigators used the Cochrane Collaboration’s tool to assess the risk of bias [19]. The included RCTs were classified as low, unclear, or high risk of bias from comprehensive evaluation from seven dimensions, including random sequence generation, allocation concealment, blinding of participants and personnel, incomplete outcome data, selective reporting, and other biases. Discrepancies were resolved with a senior research team member.

### Statistical analysis

Mean (standard deviation (SD)) was used to describe variables with normal distribution, median (IQR) was used to describe variables with skewed distribution, and number (percentage) was used to describe categorical variables.

We performed meta-analyses to compare the effects of the rehabilitation interventions with the common control group (i.e. placebo, usual care, et al.). For continuous outcomes measured by the same scale in all studies, the summary result was presented as a difference in mean (MD) with its 95% confidence intervals (CI). It estimated the amount by which the experimental intervention changed the outcome on average compared with the comparator intervention. When the studies assessed the same outcome, but measured it in a variety of ways (for example, some studies measured depression but used different psychometric scales), the standardised mean difference (SMD) with its 95% CI was used as a summary statistic [20].

*I*-square (*I*^2^) statistic was used to assess the inter-study heterogeneity: *I*^2^≤50% represented low to moderate heterogeneity, while *I*^2^≥50% represented substantial heterogeneity [20]. If there was no significant heterogeneity observed, a fixed-effects model was used, otherwise, a random-effects model, which considered variation across studies and could better deal with heterogeneity, was used to estimate the pooled effect size. Moreover, the literature review indicates that different interventions vary in rehabilitation effectiveness and importance in clinical practice [11,21–23]. Additionally, different ages and the severity of acute infection might also impact the demands and effectiveness of rehabilitation in older adults. Therefore, subgroup analyses were carried out by the intervention programmes (exercise training, respiratory rehabilitation, and others), the severity of acute infection (non-severe and severe/critical), and the mean age of participants (60–65 and >65 years old).

Egger’s test was used to assess the potential publication bias for continuous outcomes. A two-sided *P* < 0.10 was considered evidence of publication bias [24]. If publication bias was observed, sensitivity analysis was performed using the trimming and filling method to assess the effect of publication bias. If the pooled effect size and its 95% CI did not change significantly before and after trimming and filling, it suggested that the effect of publication bias was not significant and the meta-analysis results were robust [25]. All analyses were conducted in Stata version 15.0 (Stata Corp, Texas, USA).

## RESULTS

### Characteristics of included studies

**Figure 1:**
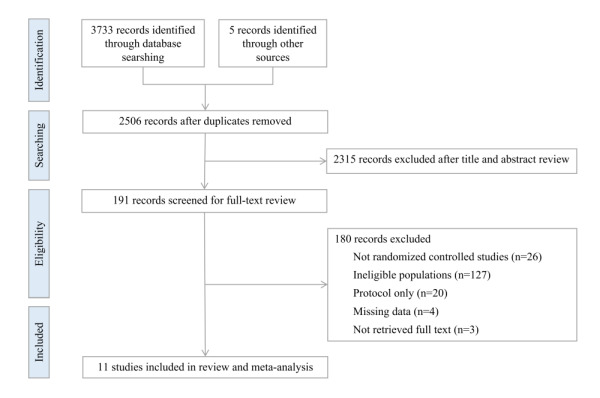
Flowchart for study selection.

In the original literature retrieval, a total of 3738 potential records were identified up to 15 December 2023. After the removal of 1232 duplicates, we carried out title and abstract screening of 2506 articles and left 191 articles screened for full-text review. Eventually, based on the exclusion and inclusion criteria, 11 articles were eligible and included in this meta-analysis and systematic review [26–36]. The literature retrieval flowchart is shown in **Figure 1**.

In brief, the majority of the included studies were conducted in Asia (two in Saudi Arabia, one in China, one in Kazakhstan, and one in Turkey) [28,29,32,34,35], three in Europe (one each in Poland, Italy, and Portugal) [26,30,36], two in Africa (all in Egypt) [27,31], and one in South America (Brazil) [33]. More details about the characteristics of the included studies are shown in Table S1 in the **Online Supplementary Document**.

### Characteristics of the participants

A total of 832 participants were included in the analysis (64.37 ± 7.94 years, 52.2% were men). 301 (36.18%) participants from 5 studies were discharged from the intensive care unit (ICU) after experiencing a severe/critical course of COVID-19 infection [26,30,33,35,36]. Two studies included 232 (27.88%) discharged patients from general wards [29,34], and four studies included 299 (35.94%) participants whose hospitalisation status was unclear [27,28,31,32]. A total of 371 (44.59%) participants from five studies were all ≥60 years old [27,28,31,32,34]. The age criteria for participants in three studies was <60 years [29,33,36], and in three studies was not specified, but the average age of all of them was >60 years [26,30,35]. The characteristics of the participants are shown in Table S1 in the **Online Supplementary Document**.

### Characteristics of rehabilitation interventions

The most common rehabilitation interventions were exercise training, including aerobic exercises (231 participants from five records) [27,28,30,32], home-based exercise training programme (50 participants from one study) [33], and exercises on the rehabilitation robot (81 participants from one study) [26]. Intervention for 210 participants from three studies was respiratory rehabilitation [34–36]. Other rehabilitation interventions included acupuncture (160 participants from one study) and photobiomodulation treatment (100 participants from one study) [29,31].

### Outcomes

#### Functional capacity

**Figure 2:**
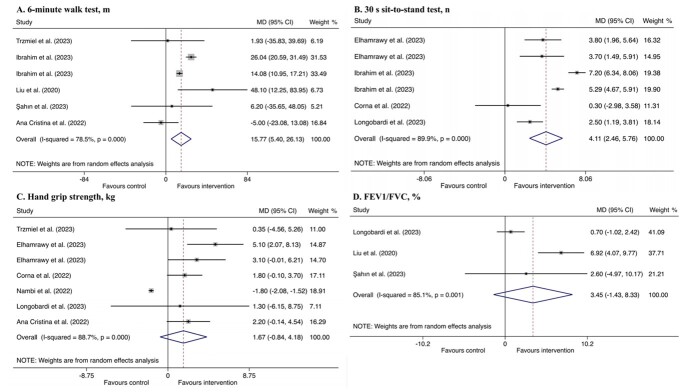
Forest plot of effects of rehabilitation interventions for old adults with long COVID in functional capacity and pulmonary function, compared to usual/standard care. **Panel A.** Effects in 6-minute walk test. **Panel B.** Effects in 30-second sit-to-stand test. **Panel C.** Effects in hand grip strength. **Panel D.** Effects in FEV1/FVC. CI – confidence interval, COVID – coronavirus disease, FEV1/FVC – forced expiratory volume in one second/forced vital capacity, MD – difference in mean.

Three outcome variables were used to assess the functional capacity (**Table 1**, **Figure 2**). Meta-analysis results showed that compared with the control groups, rehabilitation interventions significantly improved 6 MWT (MD = 15.77 m, 95% CI = 5.40, 26.13, *P* < 0.01). Subgroup analyses showed that this improvement was more pronounced in participants who received exercise training intervention (MD = 19.84 m, 95% CI = 8.13, 31.56, *P* < 0.01), experienced a non-severe course of COVID-19 infection (MD = 22.36 m, 95% CI = 10.58, 34.13, *P* < 0.01), and averaged between 60–65 years of age (MD = 19.01 m, 95% CI = 8.05, 29.96, *P* < 0.01) (Table S2 in the **Online Supplementary Document**).

**Table 1:** Comparison of treatment outcomes of rehabilitation between intervention and control groups

Outcome variables	Trials/records, No.	Participants, No.	MD (95% CI)	*P*-value	*I*^2^ (%)	*P*-heterogeneity
Functional capacity						
*6-min walk test, m*	6	387	15.77 (5.40, 26.13)	0.003	78.5	<0.001
*30 s sit-to-stand test, n*	6	250	4.11 (2.46, 5.76)	<0.001	89.9	<0.001
*Hand grip strength, kg*	7	404	1.67 (−0.84, 4.18)	0.193	88.7	<0.001
Pulmonary function						
*FEV1/FVC, %*	3	164	3.45 (−1.43, 8.33)	0.166	85.1	0.001
*Fatigue*	4	222	−0.66 (−1.13, −0.19)*	0.006	62.9	0.044
Quality of life						
*Physical functioning*	5	260	11.41 (5.59, 17.24)	<0.001	96.8	<0.001
*Bodily pain*	5	260	8.48 (4.99, 11.97)	<0.001	90.9	<0.001
*General health*	5	260	7.98 (4.29, 11.67)	<0.001	90.5	<0.001
*Role-physical*	5	260	7.13 (3.72, 10.54)	<0.001	90.2	<0.001
*Vitality*	5	260	8.19 (4.20, 12.18)	<0.001	87.4	<0.001
*Social functioning*	5	260	7.09 (3.59, 10.59)	<0.001	88.0	<0.001
*Mental health*	5	260	5.61 (2.88, 8.34)	<0.001	85.3	<0.001
*Role-emotional*	5	260	7.51 (3.15, 11.86)	0.001	90.5	<0.001
*Independence in activities of daily living*	6	516	0.31 (0.13, 0.48)*	<0.001	0.0	0.581
Depression and anxiety						
*Depression*	4	210	−0.89 (−1.76, −0.02)*	0.046	88.5	<0.001
*Anxiety*	4	210	−0.81 (−1.58, −0.05)*	0.038	85.4	<0.001

CI – confidence interval, FEV1/FVC – forced expiratory volume in one second/forced vital capacity, MD – difference in mean

*Using standard mean difference (SMD) as pooled effect size.

Rehabilitation interventions were associated with the improvement of 30 seconds STS (MD = 4.11 n, 95% CI = 2.46, 5.76, *P* < 0.001), which was more pronounced in participants with non-severe infection (MD = 5.22 n, 95% CI = 3.72, 6.72, *P* < 0.001) and who aged 60–65 years (MD = 5.05 n, 95% CI = 2.89, 7.22, *P* < 0.001) (Table S3 in the **Online Supplementary Document**).

The pooled MD of HGS was not significant (MD = 1.67 kg, 95% CI = −0.84, 4.18, *P* > 0.05). But subgroup analysis showed that rehabilitation interventions significantly improved HGS among participants with severe/critical acute infection (MD = 1.81 kg, 95% CI = 0.42, 3.19, *P* < 0.05) and aged over 65 years (MD = 2.53kg, 95% CI = 1.29, 3.78, *P* < 0.001) (Table S4 in the **Online Supplementary Document**).

#### Pulmonary function

The pooled MD of FEV1/FVC was not significant (MD = 3.45%, 95% CI = −1.43%, 8.33%, *P* > 0.05) (**Table 1**, **Figure 2**). But subgroup analysis showed that compared with control groups, rehabilitation interventions significantly improved pulmonary function among participants who received respiratory rehabilitation (MD = 6.24%, 95% CI = 3.16%, 9.32%, *P* < 0.001), with non-severe acute infection (MD = 6.92%, 95% CI = 4.07%, 9.77%, *P* < 0.001) and aged over 65 years (MD = 6.92%, 95% CI = 4.07%, 9.77%), *P* < 0.001) (Table S5 in the **Online Supplementary Document**).

#### Fatigue

**Figure 3:**
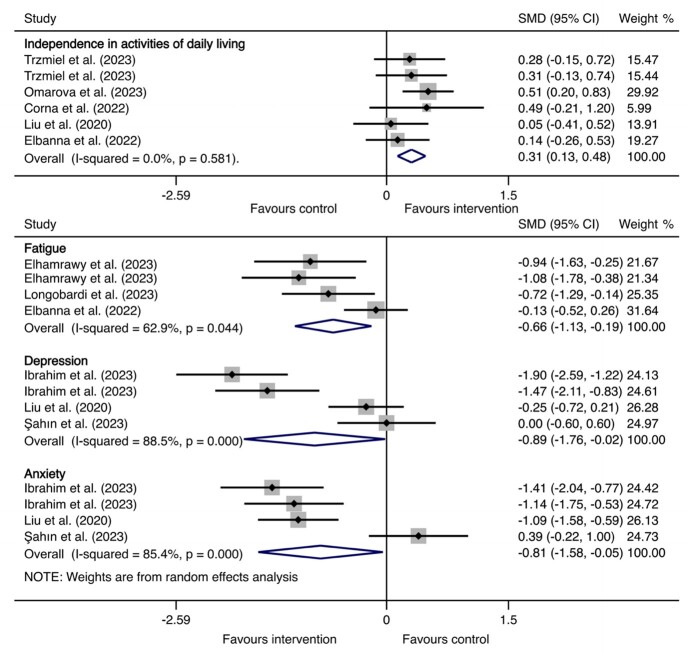
Forest plot of effects of rehabilitation interventions for old adults with long COVID in independence in activities of daily living, fatigue, depression, and anxiety, compared to usual/standard care. CI – confidence interval, COVID – coronavirus disease, SMD – standard mean difference.

Fatigue was measured by the FSS, and the higher the score, the more severe the fatigue was. Thus, decreased scores indicated improvement in fatigue. Compared with the control group, rehabilitation interventions were associated with the alleviation of fatigue (SMD = −0.66, 95% CI = −1.13, −0.19, *P* < 0.01) (**Table 1**, **Figure 3**). Subgroup analysis showed that fatigue alleviated more significantly in participants who received exercise training rehabilitation intervention, whose mean age was over 65 years, and who had severe/critical acute infection (Table S6 in the **Online Supplementary Document**).

#### Quality of life

**Figure 4:**
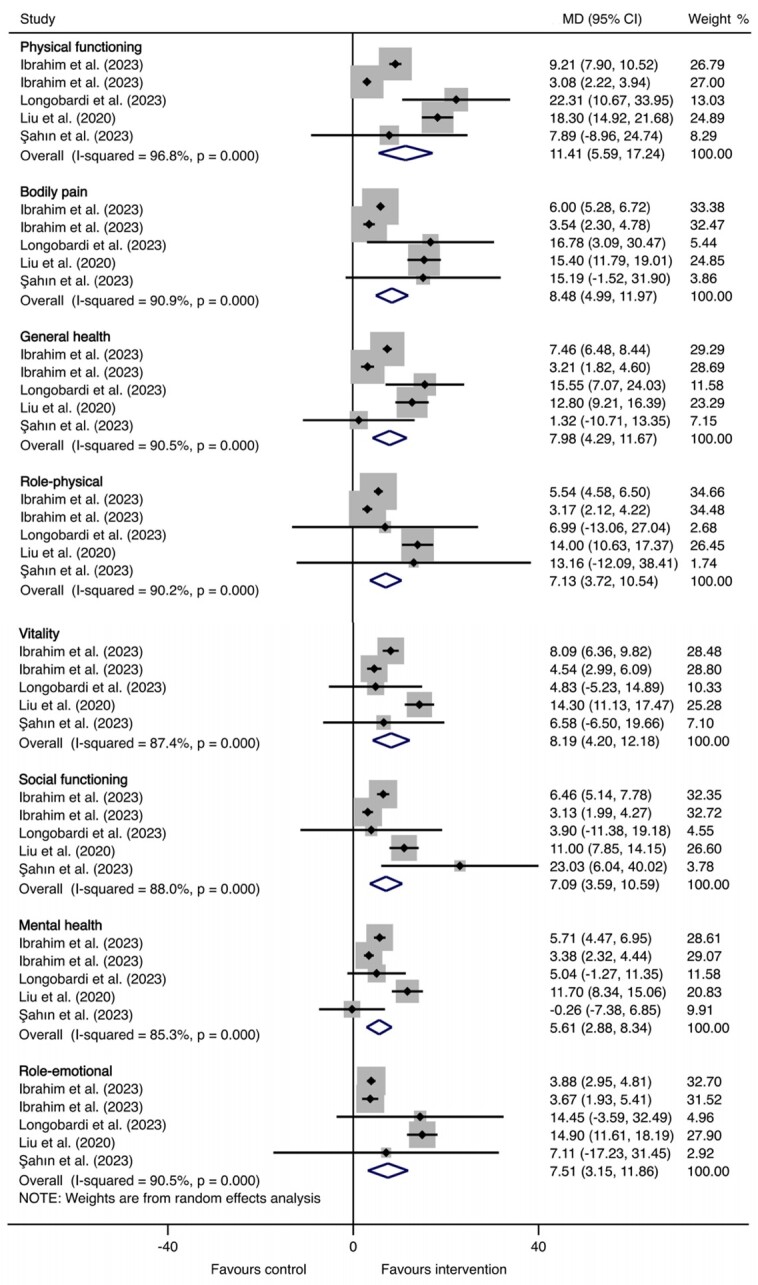
Forest plot of effects of rehabilitation interventions for old adults with long COVID in quality of life, compared to usual/standard care. CI – confidence interval, COVID – coronavirus disease, MD – difference in mean.

Results of meta-analysis suggested that compared to the control group, participants in the rehabilitation interventions group showed significant improvement in all domains of the SF-36: physical functioning (MD = 11.41 points, 95% CI = 5.59, 17.24, on the 100-point SF-36 subscale for physical functioning), bodily pain (MD = 8.48 points, 95% CI = 4.99, 11.97, on the 100-point SF-36 subscale for bodily pain), general health (MD = 7.98 points, 95% CI = 4.29, 11.67, on the 100-point SF-36 subscale for general health perception), role-physical (MD = 7.13 points, 95% CI = 3.72, 10.54, on the 100-point SF-36 subscale for role limitation due to physical health), vitality (MD = 8.19 points, 95% CI = 4.20, 12.18, on the 100-point SF-36 subscale for vitality), social functioning (MD = 7.09 points, 95% CI = 3.59, 10.59, on the 100-point SF-36 subscale for social functioning), mental health (MD = 5.61 points, 95% CI = 2.88, 8.34, on the 100-point SF-36 subscale for perceived mental health), and role-emotional (MD = 7.51 points, 95% CI = 3.15, 11.86, on the 100-point SF-36 subscale for role limitation due to mental health), all *P*-value <0.01 (**Table 1**, **Figure 4**).

Subgroup analyses showed that the respiratory rehabilitation intervention group improved better than the exercise training intervention group in all domains of quality of life, and the mean age >65 group was better than the 60–65 group. In contrast, improvements in all domains of quality of life varied across severity in the acute infection phase. More details about the results of subgroup analyses are shown in Table S7 in the **Online Supplementary Document**.

#### Independence in activities of daily living, depression, and anxiety

Compared with the control group, rehabilitation interventions were associated with the improvement of independence in activities of daily living (SMD = 0.31, 95% CI = 0.13, 0.48, *P* < 0.001) (**Table 1**, **Figure 3**). Depression and anxiety were both measured by different psychometric scales and the higher the score, the more severe the anxiety/depression was. Thus, decreased scores indicated alleviation of anxiety/depression. Relief was observed in both depression (SMD = −0.89, 95% CI = −1.76, −0.02, *P* < 0.05) and anxiety (SMD = −0.81, 95% CI = −1.58, −0.05, *P* < 0.05) for participants in the rehabilitation intervention group compared to the comparison group (**Table 1**). Subgroup analysis showed that both anxiety and depression alleviated more significantly in participants who received exercise training rehabilitation intervention and those with non-severe acute infection (Table S8–9 in the **Online Supplementary Document**).

### Risk of bias

**Figure 5:**
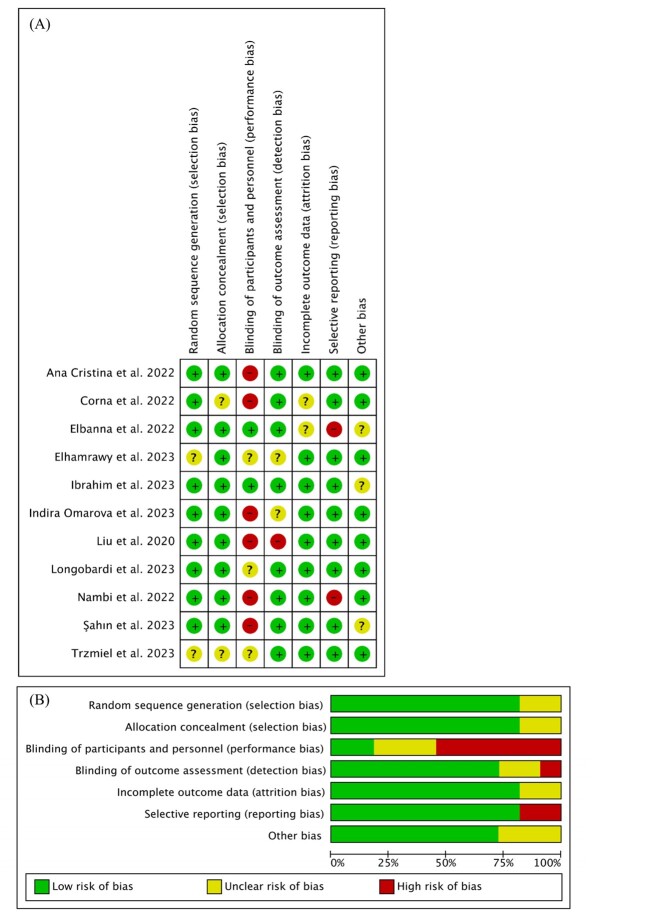
Risk of bias. **Panel A.** Risk of bias summary: review authors’ judgements about each risk of bias item for each included study. **Panel B.** Risk of bias graph: review authors’ judgements about each risk of bias item presented as percentages across all included studies.

**Figure 5** summarises the risk of bias of included studies based on the Cochrane criteria. For performance bias, six studies were graded as having a high risk, as the personnel and participants were not blinded. One study was graded as having a high risk of detection bias because the outcome assessment was not blinded. Two studies were graded as having a high risk of reporting bias because of selective reporting. Overall, the risk of all types of bias was low except for performance bias.

The results of Egger’s test showed that publication bias was not observed for all outcome variables (*P* > 0.1) except HGS and fatigue (*P* < 0.1) (Table S10 in the **Online Supplementary Document**). Sensitivity analysis by trimming and filling method showed that the MD of HGS was 1.67 kg (95% CI = −0.84, 4.18) before and 0.22 kg (95% CI = 0.03, 1.77) after trimming and filling, and the SMD of fatigue was −0.66 (95% CI = −1.13, 0.19) both before and after trimming and filling. The pooled effect sizes and their 95% CIs did not change significantly (Table S11 in the **Online Supplementary Document**). The application of the trimming and filling method demonstrated that the effect sizes for HGS and fatigue remained relatively stable after accounting for potential publication bias, which suggested that the observed publication bias had a minimal impact and the meta-analysis results are robust and reliable.

## DISCUSSION

This systematic review and meta-analysis of the existing RCTs aimed to comprehensively summarise the pattern and effectiveness of current rehabilitation interventions for old adults aged over 60 years with long COVID. A total of 11 studies involving 832 participants (64.37 ± 7.94 years old) were included in the analysis. Our results showed that old adults who underwent rehabilitation interventions experienced great improvement in functional capacity, quality of life, independence in activities of daily living, and alleviation in fatigue, depression and anxiety outcomes compared with those who received usual or standard care. To our knowledge, up to now, this study is the first systematic review and meta-analysis of RCTs in this important field. By consolidating the available evidence, this study will provide valuable evidence-based information for health care professionals, policymakers, and researchers to make informed decisions regarding implementing effective rehabilitation strategies for old adults with long COVID. Ultimately, the findings of this study would help improve the care and outcomes for this vulnerable population, and contribute to their overall health and well-being, thereby reducing the burden on medical resources and the health care system.

Regarding the components of rehabilitation programmes for old adults, the results of this review showed that the most common was exercise training with aerobic exercise [27,28,30,32,33], followed by pulmonary rehabilitation programmes including respiratory muscle training, cough exercise, diaphragmatic training, stretching exercise, and home exercise, etc. [34–36]. As some patients experienced muscle strength loss after discharge from the hospital, rehabilitation robot-assisted exercise training has also been observed to be effective [26]. Some rehabilitation measures were outside the scope of our review, such as nutraceuticals and dietary supplements, and Tosato et al. suggested that bioactive foods, supplements, and nutraceuticals might be used for the management of long-term COVID-19 clinical sequelae [37].

Our results are consistent with the findings of several previous studies reviewing the general population, in which rehabilitation interventions significantly improved the functional capacity [9,11,38–40], fatigue [40], quality of life [9,38–40], and mental health [38,40] of patients with long COVID compared with usual care, but differ in that we focused on a special group of older adults. In Torres et al.’s meta-analysis (age range: 18–75 years old), there was a significant difference in the 6 MWT (MD = 51.69 m, 95% CI = 36.99, 66.38, *P* < 0.001) [11], whereas this value was 15.77 m (95% CI = 5.40, 26.13, *P* < 0.01) in our study, suggesting that rehabilitation interventions might be more effective in younger people. The relationship between rehabilitation interventions and improvement in pulmonary function is highly heterogeneous across reviews [9,11,38,39,41,42]. In this study, we found no difference between rehabilitation interventions and standard/usual care in pulmonary function assessed by the FEV1/FVC (MD = 3.45%, 95% CI = −1.43%, 8.33%, *P* > 0.05), which is in line with the review results of by Pouliopoulou et al. (median (IQR) age = 50 (47–56) years; assessed by FEV1 and FVC) [9]. In addition, non-significant results for pulmonary function improvement were also observed in a review conducted by AL-Mhanna and his colleagues targeting pulmonary rehabilitation among the general population [41]. However, in Torres et al.’s review (age range: 18–75 years), rehabilitation interventions improved pulmonary function (assessed by FEV1 (MD = 3.49%, 95% CI = 1.25%, 5.73%, *P* = 0.002)) significantly compared with the control group [11], the same as a meta-analysis among general age of Yang et al. [43]. Therefore, the results should also be interpreted with caution as different reviews were of variable quality and included literature with short follow-up or low quality.

Subgroup analyses showed that in terms of functional capacity, exercise training was more significant in improving the 6 MWT, whereas pulmonary rehabilitation was more effective in improving HGS. It is suggested that different interventions such as exercise training and pulmonary rehabilitation should be combined to improve functional capacity more comprehensively in older adults with long COVID. In addition, we observed better improvement of functional capacity in old adults who were non-severe during acute infection and aged 60–65 years, which is in accordance with the expected results. Regarding lung function, better improvement was observed in older adults who received pulmonary rehabilitation, were non-severe during acute infections, and were older than 65 years old. In addition, quality of life was improved more significantly in older adults who received pulmonary rehabilitation, had severe/critical acute infections, and were older than 65 years old. This indicates that rehabilitation interventions, especially pulmonary rehabilitation, are effective in improving patients’ quality of life and organic function even in the presence of severe/critical infections or older age, which further emphasises the importance of rehabilitation interventions for older adults with long COVID.

Because of the multi-organ involvement and diverse presentation of COVID-19 as well as age-related issues like frailty, cognitive impairments, and multimorbidity, treating older post-acute COVID-19 patients is highly challenging [12]. Multidisciplinary rehabilitation plays an important role in the older population, whether it is for COVID-19 or other disorders such as debilitation [12,44–46]. The European Geriatric Medicine Society (EuGMS) provided detailed guidance on the management of post-acute COVID-19 patients in geriatric rehabilitation, advising personalised treatment regimes for older patients through multidisciplinary rehabilitation [12]. A review by McCarthy et al. showed that multidisciplinary team rehabilitation for older adults with COVID-19 in acute or post-acute inpatient hospital settings resulted in significant improvement in function [10]. An interdisciplinary team could include Physicians, Nurses, Physiotherapists, Occupational Therapists, Dietitians, Speech and Language Therapists, Psychologists, Social Workers, etc., who play their respective roles [12]. For example, Physicians evaluate patients’ physical conditions and manage symptoms; Physiotherapists develop and guide physical training programmes to help improve muscle strength and motor function; Occupational therapists assist in restoring ability to daily living activities, such as the recommendation and use of assistive devices; Respiratory Therapists provide respiratory training guidance to improve respiratory function; Dietitians develop nutritional plans to manage weight loss, muscle wasting, and overall nutritional conditions; Psychologists provide psychological support and treatment to address psychological problems such as anxiety and depression; and Social workers help navigate social services, support systems, and provide resources to patients and their families [12]. Through the collaboration of an interdisciplinary team, a personalised treatment regime will be formulated and adjusted promptly according to the condition of patient. In addition, in the process of multidisciplinary rehabilitation, publicity and education for patients and their family members should be strengthened, and active family participation and community support should also be encouraged, such as the establishment of family support groups [47]. Since health care resources are often limited and strained, it is expected to explore more accessible and cost-effective rehabilitation programmes in the older population under the guidance of a multidisciplinary team, to alleviate the pressure on medical resources. In addition, due to the vulnerability of the older population, we thus recommend that any rehabilitation intervention should follow the principle of safety to avoid additional injuries to them.

This study included RCTs from multiple countries and regions around the world, and such geographic diversity enhances the broad applicability of the findings. However, cultural contexts and health care system differences in different regions may affect the implementation and effectiveness of rehabilitation interventions for old adults with long COVID. For one thing, cultural beliefs and attitudes towards rehabilitation and health care might influence old adults’ participation and treatment adherence in different cultural contexts [48,49]. For another, the availability and level of medical resources, as well as the structure of health care systems, vary from region to region, which might also affect the type, intensity and outcome of rehabilitation interventions [50]. For example, developed countries usually have better health care infrastructure and resources, with higher standards and quality of rehabilitation services, making it easier for older adults to access specialised rehabilitation services. While in some developing countries, medical resources might be mainly concentrated in cities and less in rural areas, which might lead to disparities in rehabilitation outcomes between urban and rural areas [51]. Therefore, future research on rehabilitation interventions for older adults with long COVID should further consider the impact of cultural and health care system differences to ensure the effectiveness and applicability of interventions.

### Strengths and limitations

Although several systematic reviews or meta-analyses of rehabilitation for patients with long COVID have emerged, the limitations of the currently published evidence are the lack of analyses of the specific population of the elderly as they focused primarily on the general population [9,11,38,39,41–43,52], and most included studies of which were observational studies [53]. To our knowledge, this study is the first systematic review and meta-analysis of rehabilitation interventions for old patients with long COVID which only included RCTs. Our review identified eleven randomised controlled trials of a wide range of common rehabilitation measures, such as exercise training and respiratory rehabilitation, and assessed a full range of outcomes. As in previous reviews [10,11,43,53], heterogeneity between studies/trials was unavoidable, so we conducted subgroup analyses to explore sources of heterogeneity and to compare the effects of different rehabilitation interventions, as well as the effectiveness of rehabilitation interventions in old populations with different ages and acute phase severity. The low risk of bias and very little publication bias further supported the robustness and accuracy of our results. In addition, we used a comprehensive search strategy and searched all relevant sources to retrieve all potential eligible randomised clinical trials. Therefore, this study could provide valuable high-quality evidence for the field of rehabilitation interventions for old adults with long COVID.

However, there are also some limitations to this study. First, because of the very limited RCTs examining rehabilitation interventions in older populations, we used the restriction of “median or mean age greater than 60 years” as a definition and inclusion criterion for older populations to capture more available studies. Some participants younger than 60 years old might have been included in the analyses, which might impact our results to some extent. Therefore, we performed subgroup analyses of participants’ ages to assess the robustness of the results. Future RCTs of rehabilitation of long COVID targeting older adults, a high-risk population, should be designed to provide more high-quality evidence. In addition, comorbidity is an important consideration for rehabilitation outcomes in older adults, we summarised the characteristics of comorbidity of the included participants but did not analyse this factor due to the limitation of original data, suggesting that future research should further explore the impact of comorbidity on the effectiveness of rehabilitation interventions in older adults to better guide the clinical practice. Furthermore, due to the specific features of rehabilitation intervention programmes, it was difficult to blind study participants, whose expectations might affect the perception of intervention effects. So performance bias was unavoidable and might lead to an overestimation of the true effects of the rehabilitation interventions. To address this bias, future research could use more objective outcome measures, thereby mitigating the impact of participants’ expectations on outcomes. Also, blinding of therapists and outcome assessors could help control bias. Finally, given the complexity of long COVID, it is critical to explore all potential rehabilitation programmes including medicine intervention. However, none of the included studies researched this programme among older adults, which highlights a gap in the current research. Future research should pay attention to integrating medicinal interventions, such as anti-inflammatory drugs and supplements, to evaluate their effectiveness in combination with other interventions for old adults with long COVID [54,55]. Such a comprehensive approach could provide deeper insights into the best practices for managing long COVID in older adults and help to develop more effective, multifaceted rehabilitation programmes. In summary, it is emphasised that there is an urgent need for RCTs with larger sample sizes, rigorous methodologies and comprehensive interventions to address the knowledge gaps mentioned above among old adults with long COVID in future research.

### Implications for clinical practice and policy-making

These results highlight the importance of effective screening programmes to detect long COVID timely among old adults. Early referral to a multidisciplinary rehabilitation team to develop a personalised rehabilitation plan based on the physical condition and specific demands of the old adults, with regular assessments and adjustments, to maximise the effectiveness of the intervention. Besides, training and education of health care workers should be strengthened to draw their attention to long COVID and to understand the best practices for managing long COVID in old adults. Furthermore, comorbidities might not only increase the risk of developing long COVID in older adults, but also affect rehabilitation outcomes by increasing medical complexity and demands of rehabilitation [56–58]. Among older adults long COVID, the effectiveness of rehabilitation interventions might be somewhat restricted due to comorbidities, suggesting that individual comorbidities should be taken into account in clinical practice and further research.

For policy-making, it is important to further support research on the rehabilitation of old adults with long COVID by ensuring the allocation of adequate funding and resources and encouraging the exploration of innovative rehabilitation methods. Furthermore, there is a necessity to formulate national or international rehabilitation guidelines to provide standardised care and management guidance for old adults with long COVID. Finally, public health campaigns should also be conducted to increase public awareness of long COVID rehabilitation.

## CONCLUSIONS

Long COVID is of particular concern among old adults. Our finding showed that over-60-year-old adults with long COVID who underwent rehabilitation interventions experienced great improvement in functional capacity, quality of life, independence in activities of daily living, and alleviation in fatigue, depression and anxiety outcomes compared with those who received usual or standard care. These findings provide valuable implications for clinical practice and policy-making, suggesting that screening and management of long COVID among older adults should be strengthened. Formulating individualised rehabilitation programmes through multidisciplinary management to improve the complete health status and functional status of older patients, thereby reducing the long-term disease burden caused by long COVID and fostering healthy aging during the post-pandemic era.

## Additional material


Online Supplementary Document
