# Supplementary material for: Effects of rehabilitation interventions for old adults with long COVID: A systematic review and meta-analysis of randomised controlled trials
Source: J Glob Health. 2024 Sep 6;14:05025. doi: 10.7189/jogh.14.05025 (PMC11377967; doi:10.7189/jogh.14.05025)
Supplement: Online Supplementary Document [file jogh-14-05025-s001.pdf]

ONLINE SUPPLEMENTARY DOCUMENT

Title: Effects of rehabilitation interventions for old adults with long COVID: A systematic review and meta-analysis of randomized controlled trials

Authors: Jie Deng, Chenyuan Qin, Minjung Lee, Yubin Lee, Myoungsoon You, Jue Liu

Content	Page
**Appendix 1.** Search strategy	2
**Appendix 2.** PRISMA 2020 Checklist	3-5
**Table S1.** Characteristics of the included studies.	6-8
**Table S2.** Subgroup analysis of 6-minute walk test by mean age, intervention, and severity in acute infection.	9
**Table S3.** Subgroup analysis of 30s sit-to-stand test by mean age and severity in acute infection.	9
**Table S4.** Subgroup analysis of hand grip strength by mean age, intervention and severity in acute infection.	9
**Table S5.** Subgroup analysis of pulmonary function (FEV1/FVC, %) by mean age, intervention and severity in acute infection.	10
**Table S6.** Subgroup analysis of fatigue by mean age, intervention and severity in acute infection.	10
**Table S7.** Subgroup analysis of quality of life by mean age, intervention and severity in acute infection.	11-13
**Table S8.** Subgroup analysis of anxiety by mean age, intervention and severity in acute infection.	13
**Table S9.** Subgroup analysis of depression by mean age, intervention and severity in acute infection.	13
**Table S10.** Publication bias.	14
**Table S11.** Sensitivity analysis by trimming and filling method.	14

Appendix 1. Search strategy

PubMed	(((Post-Acute COVID-19 Syndrome[MeSH Terms]) OR (long covid[Title/Abstract]) OR (post covid[Title/Abstract]) OR long-covid[Title/Abstract] OR post-covid[Title/Abstract]) OR ((long-term[Title/Abstract] OR post-acute[Title/Abstract] OR sequela*[Title/Abstract] OR sequala*[Title/Abstract]) AND (SARS-CoV-2[MeSH Terms] OR COVID-19[MeSH Terms] OR SARS-CoV-2[Title/Abstract] OR COVID-19[Title/Abstract] OR (COVID 19[Title/Abstract])))) AND ((randomized controlled trial[MeSH Terms]) OR (randomized control* trial[Title/Abstract]) OR (clinical trial[Title/Abstract]) OR (randomised control* trial[Title/Abstract]) OR intervention[Title/Abstract] OR RCT[Title/Abstract]) AND (rehabilitation[MeSH Terms] OR rehabilitation[Title/Abstract] OR recover*[Title/Abstract] OR recuperat*[Title/Abstract] OR management[Title/Abstract] OR telehealth[Title/Abstract] OR exercise[Title/Abstract] OR training[Title/Abstract] OR therapy[Title/Abstract] OR medicine[Title/Abstract] OR physical[Title/Abstract])
Embase	(('long covid'/exp OR 'long covid':ti,ab,kw OR 'post covid':ti,ab,kw) OR (('long term':ti,ab,kw OR 'post acute':ti,ab,kw OR sequela*:ti,ab,kw OR sequala*:ti,ab,kw) AND ('sars cov 2':ti,ab,kw OR 'covid 19':ti,ab,kw OR 'sars cov 2'/exp OR 'covid 19'/exp))) AND (rehabilitation/exp OR rehabilitation:ti,ab,kw OR recover*:ti,ab,kw OR recuperat*:ti,ab,kw OR management:ti,ab,kw OR telehealth:ti,ab,kw OR exercise:ti,ab,kw OR training:ti,ab,kw OR therapy:ti,ab,kw OR medicine:ti,ab,kw OR physical:ti,ab,kw) AND ('randomized controlled trial'/exp OR 'randomized control* trial':ti,ab,kw OR 'clinical trial':ti,ab,kw OR 'randomised control* trial':ti,ab,kw OR intervention:ti,ab,kw OR rct:ti,ab,kw)
Web of Science	TS=((long covid) OR (post covid) OR long-covid OR post-covid OR ((long-term OR post-acute OR sequela* OR sequala*) AND (SARS-CoV-2 OR COVID-19 OR (COVID 19)))) AND TS=(rehabilitation OR recover* OR recuperat* OR management OR telehealth OR exercise OR training OR therapy OR medicine OR physical) AND TS=((randomized control* trial) OR (clinical trial) OR (randomised control* trial) OR intervention OR RCT)
Cochrane Library	#1 (rehabilitation OR recover* OR recuperat* OR management OR telehealth OR exercise OR training OR therapy OR medicine OR physical OR intervention):ti,ab,kw<br>#2 MeSH descriptor: [Rehabilitation] explode all trees<br>#3 ((long covid) OR (post covid) OR ((long-term OR post-acute OR sequela* OR sequala*) AND (SARS-CoV-2 OR COVID-19))):ti,ab,kw<br>#4 MeSH descriptor: [Post-Acute COVID-19 Syndrome] explode all trees<br>#5 ((randomized control* trial) OR (randomised control* trial) OR intervention OR (clinical trial) OR RCT):ti,ab,kw<br>#6 MeSH descriptor: [Randomized Controlled Trial] explode all trees<br>#7 (old OR elder OR elderly OR older OR senior):ti,ab,kw<br>#8 #1 OR #2<br>#9 #3 OR #4<br>#10 #5 OR #6<br>#11 #8 AND #9 AND #10<br>#12 #7 AND #11
Scopus	TITLE-ABS-KEY ("long covid" OR "post covid" OR long-covid OR post-covid OR ((long-term OR post-acute OR sequela* OR sequala*) AND (sars-cov-2 OR covid-19))) AND TITLE-ABS-KEY (rehabilitation OR recovery OR recuperat* OR management OR telehealth OR exercise OR training OR therapy OR medicine OR physical) AND TITLE-ABS-KEY ("randomized controlled trial" OR "randomised controlled trial" OR "randomized control* study" OR "randomised control* study" OR "clinical trial" OR intervention OR RCT)

Appendix 2. PRISMA 2020 Checklist

Section and Topic	Item #	Checklist item	Location where item is reported
**TITLE**			
Title	1	Identify the report as a systematic review.	P1, 2
**ABSTRACT**			
Abstract	2	See the PRISMA 2020 for Abstracts checklist.	P2
**INTRODUCTION**			
Rationale	3	Describe the rationale for the review in the context of existing knowledge.	P3
Objectives	4	Provide an explicit statement of the objective(s) or question(s) the review addresses.	P4
**METHODS**			
Eligibility criteria	5	Specify the inclusion and exclusion criteria for the review and how studies were grouped for the syntheses.	P4
Information sources	6	Specify all databases, registers, websites, organisations, reference lists and other sources searched or consulted to identify studies. Specify the date when each source was last searched or consulted.	P4
Search strategy	7	Present the full search strategies for all databases, registers and websites, including any filters and limits used.	P4
Selection process	8	Specify the methods used to decide whether a study met the inclusion criteria of the review, including how many reviewers screened each record and each report retrieved, whether they worked independently, and if applicable, details of automation tools used in the process.	P4
Data collection process	9	Specify the methods used to collect data from reports, including how many reviewers collected data from each report, whether they worked independently, any processes for obtaining or confirming data from study investigators, and if applicable, details of automation tools used in the process.	P4
Data items	10a	List and define all outcomes for which data were sought. Specify whether all results that were compatible with each outcome domain in each study were sought (e.g. for all measures, time points, analyses), and if not, the methods used to decide which results to collect.	P4, 5
	10b	List and define all other variables for which data were sought (e.g. participant and intervention characteristics, funding sources). Describe any assumptions made about any missing or unclear information.	P4
Study risk of bias assessment	11	Specify the methods used to assess risk of bias in the included studies, including details of the tool(s) used, how many reviewers assessed each study and whether they worked independently, and if applicable, details of automation tools used in the process.	P5
Effect measures	12	Specify for each outcome the effect measure(s) (e.g. risk ratio, mean difference) used in the synthesis or presentation of results.	P5

Section and Topic	Item #	Checklist item	Location where item is reported
Synthesis methods	13a	Describe the processes used to decide which studies were eligible for each synthesis (e.g. tabulating the study intervention characteristics and comparing against the planned groups for each synthesis (item #5)).	P5
	13b	Describe any methods required to prepare the data for presentation or synthesis, such as handling of missing summary statistics, or data conversions.	P5
	13c	Describe any methods used to tabulate or visually display results of individual studies and syntheses.	P5
	13d	Describe any methods used to synthesize results and provide a rationale for the choice(s). If meta-analysis was performed, describe the model(s), method(s) to identify the presence and extent of statistical heterogeneity, and software package(s) used.	P5
	13e	Describe any methods used to explore possible causes of heterogeneity among study results (e.g. subgroup analysis, meta-regression).	P5
	13f	Describe any sensitivity analyses conducted to assess robustness of the synthesized results.	P5, 6
Reporting bias assessment	14	Describe any methods used to assess risk of bias due to missing results in a synthesis (arising from reporting biases).	P5
Certainty assessment	15	Describe any methods used to assess certainty (or confidence) in the body of evidence for an outcome.	P5, 6
**RESULTS**			
Study selection	16a	Describe the results of the search and selection process, from the number of records identified in the search to the number of studies included in the review, ideally using a flow diagram.	P6,19
	16b	Cite studies that might appear to meet the inclusion criteria, but which were excluded, and explain why they were excluded.	P6, 19
Study characteristics	17	Cite each included study and present its characteristics.	P6, Table S1
Risk of bias in studies	18	Present assessments of risk of bias for each included study.	P8, 23
Results of individual studies	19	For all outcomes, present, for each study: (a) summary statistics for each group (where appropriate) and (b) an effect estimate and its precision (e.g. confidence/credible interval), ideally using structured tables or plots.	P18, 20-22
Results of syntheses	20a	For each synthesis, briefly summarise the characteristics and risk of bias among contributing studies.	P6-8, 20-22
	20b	Present results of all statistical syntheses conducted. If meta-analysis was done, present for each the summary estimate and its precision (e.g. confidence/credible interval) and measures of statistical heterogeneity. If comparing groups, describe the direction of the effect.	P6-8, 20-22
	20c	Present results of all investigations of possible causes of heterogeneity among study results.	Table S2-9
	20d	Present results of all sensitivity analyses conducted to assess the robustness of the synthesized results.	Table S2-9, 11

Section and Topic	Item #	Checklist item	Location where item is reported
Reporting biases	21	Present assessments of risk of bias due to missing results (arising from reporting biases) for each synthesis assessed.	P8, Table S10
Certainty of evidence	22	Present assessments of certainty (or confidence) in the body of evidence for each outcome assessed.	P18
**DISCUSSION**			
Discussion	23a	Provide a general interpretation of the results in the context of other evidence.	P9-12
	23b	Discuss any limitations of the evidence included in the review.	P11-12
	23c	Discuss any limitations of the review processes used.	P11-12
	23d	Discuss implications of the results for practice, policy, and future research.	P12
**OTHER INFORMATION**			
Registration and protocol	24a	Provide registration information for the review, including register name and registration number, or state that the review was not registered.	P4
	24b	Indicate where the review protocol can be accessed, or state that a protocol was not prepared.	P4
	24c	Describe and explain any amendments to information provided at registration or in the protocol.	NA
Support	25	Describe sources of financial or non-financial support for the review, and the role of the funders or sponsors in the review.	P13
Competing interests	26	Declare any competing interests of review authors.	P13
Availability of data, code and other materials	27	Report which of the following are publicly available and where they can be found: template data collection forms; data extracted from included studies; data used for all analyses; analytic code; any other materials used in the review.	P13

Table S1. Characteristics of the included studies.

Authors	Year	Country	Study time	Population	Participants, No.	Age, mean ±SD, y	Male, No. (%)	Comorbidity, No. (%)	Severity in acute infection, No. (%)	Length of inpatient stay, mean±SD/median (IQR), days	Months of post COVID-19, mean±SD	Intervention	Control	Outcomes
Elhamrawy et al.	2023	Egypt	2022.09-2023.01	Participants aged ≥60 years, infected with COVID-19 with mild-to-moderate symptoms, and at least 3 months post-recovery.	54	66.07±3.74	35 (64.8)	NA	Mild-to-moderate: 54 (100)	NA	7.3±2.1 months	Tai Chi: four 60-minute sessions of Tai Chi exercises weekly for 12 weeks.<br>Aerobic Training: four supervised 60-minute aerobic training sessions weekly for 12 weeks.	Maintain usual activities of daily living	HGS, fatigue, and physical performance by the Senior Fitness Test
Trzmiel et al.	2023	Poland	2022.01-2022.08	Hospitalized patients with post-COVID-19 fatigue syndrome.	81	66.20±10.18	NA	NA	Severe: 81 (100)	NA	NA	Standard neurological rehabilitation for 75 min a day, complemented by 45 minutes of exercises on the rehabilitation robot	Standard neurological rehabilitation for 120 min a day	Functional physical ability (Berg scale, Tinetti scale, 6MWT), muscle strength (isokinetic muscle force of elbow flexors and extensors, HGS), and independence in activities of daily living (BI, FIM)
Ibrahim et al.	2023	Saudi Arabia	2022.03-2022.08	Post-covid-19 older subjects, aged 60-80 years.	72	62.6±4.6	31 (43.1)	Heart disease: 60 (83.3), dyslipidemia: 63 (87.5), type 2 diabetes mellitus: 60 (83.3), cancer: 8 (11.1), chronic inflammatory lung disease: 52 (72.2)	Mild illness: 17 (23.6), pneumonia: 28 (38.9), severe pneumonia: 27 (37.5)	NA	NA	Moderate-intensity aerobic exercises: walking on treadmill for 20 minutes at 50-70% of the maximum heart rate, 4 times per week for 10 weeks.<br>Low-intensity aerobic exercises: walking on treadmill for 20 minutes at 40-50% of the maximum heart rate, 4 times per week for 10 weeks.	Medical care and advice	Primary outcomes: functional exercise capacity (6-MWT), and functional state and independence of patients after COVID-19 infection (PCFS); Secondary outcomes: functional capacity of the lower limb muscle (1-min STS test), quality of life (SF-36), anxiety and depression (HADS)
Omarova et al.	2023	Kazakhstan	2022.03-2022.07	Patients older than 18 years with post COVID-19 condition.	160	61.3±4.26 (31-83)	39 (24.4)	Chronic bronchitis: 143 (89.4), arterial hypertension: 82 (51.2), coronary heart disease: 76 (47.5), diseases of the motor system: 33 (20.6), diabetes mellitus: 22	NA	13.2±1.99	NA	Complex rehabilitation methods with acupuncture	Complex rehabilitation methods	Modified Medical Research Council Dyspnea Scale, BI, 6 MWT, and the Borg scale

								(13.7), cerebrovascular diseases: 6 (3.7)						
Corna et al.	2022	Italy	2021.10-2022.09	Patients with subacute COVID-19, with defined diagnosis of interstitial pneumonia due to COVID-19 before.	32	70.55±10.63	19 (59.4)	NA	Critical: 8 (25.0), severe: 24 (75.0)	6.85±13.22 (length in ICU)	NA	Standard inpatient rehabilitation program and aerobic training (performed with an arm crank ergometer for 30 min/day, 5 days/week, for a total of 10 sessions)	Standard inpatient rehabilitation program	Primary outcome: feasibility of the intervention. Secondary outcome: the TUG test, the muscle torque of the knee extensors of both legs, handgrip test, 30 s STS test, CAS-I, and FIM
Nambi et al.	2022	Saudi Arabia	2020.03-2021.04	Men in the age range of 60–80 years with post-COVID-19 Sarcopenia.	73	63.65±3.16	73 (100.0)	NA	NA	NA	NA	High-intensity aerobic training and strength training for eight weeks	Low-intensity aerobic training and strength training for eight weeks	HGS, muscle quantity, kinesiophobia, and quality of life
Longobardi et al.	2023	Brazil	2020.11-2022.04	Severe/critical COVID-19 survivors aged 45 years or older, discharged from the intensive care unit (ICU) 3-6 months ago.	50	61±7.33	25 (50.0)	Hypertension: 28 (56.0), dyslipidemia: 27 (54.0), rheumatic disease: 16 (32.0), diabetes mellitus: 18 (36.0), cardiovascular disease: 10 (20.0), psychological disease: 10 (20.0), pulmonary disease: 8 (16.0), hypothyroidism: 9 (18.0), others: 5 (10.0)	severe: 12 (24.0), critical: 38 (76.0)	home-based exercise training group: 18 (13), control group: 19 (12)	158.5±33.7 days	16-week, 3-times-a-week (~60–80 min/session), semi-supervised, home-based exercise training programm	Usual care	Health-related quality of life, cardiorespiratory fitness and pulmonary function, functional capacity and muscle strength, anthropometry and body composition, laboratory analysis, persistent symptoms, and physical activity level
Ana Cristina et al.	2022	Portugal	NA	Adult patients (aged ⩾18 years) with respiratory insufficiency due to COVID-19 hospitalized at the ICU.	96	67.47±13.32	41 (42.7)	Heart failure: 9 (9.4), hypertension: 16 (16.7), arrhythmia: 6 (6.3), myocardial infarction: 4 (4.2), diabetes: 8 (8.3), dyslipidemia: 5 (5.2), stroke: 6 (6.3), thyroid disease: 2 (2.1), kidney disease: 4 (4.2), malignancy: 19 (19.8)	NA	14.25±6.38	NA	Functional and respiratory multidisciplinary rehabilitation programme, 15-30 min per session, twice per day, 6 days per week, until 12 weeks after discharge.	Usual care	Primary outcome: functional capacity (6MWT). Secondary outcome: Borg Rating of Perceived Exertion, Medical Research Council sum-score and HGS.

Liu et al.	2020	China	NA	Hospitalized elderly patients due to COVID-19 aged 65 years or above.	72	69.15 ±7.75	49 (68.1)	Hypertension: 18 (25.0), type 2 diabetes mellitus: 18 (25.0), osteoporosis: 14 (19.4)	NA	NA	NA	Respiratory rehabilitation: respiratory muscle training, cough exercise, diaphragmatic training, stretching exercise, and home exercise	Usual care	Primary Outcome: respiratory function; Secondary Outcome: exercise endurance (6MWT), activities of daily living and quality of life, psychological status assessment (anxiety and depression scores).
Şahın et al.	2023	Turkey	2021.02-2021.07	Hospitalized COVID-19 survivors with post-COVID-19 condition (ICU and ward for >10 days)	42	60.67 ±8.62	28 (66.7)	Presence of comorbidity: 27 (64.3)	NA	Study group: 12 (5-15), control group: 11 (8-14)	NA	Home-based pulmonary rehabilitation program with telecoaching: breathing exercises, strength training, and regular walking program	Home-based pulmonary rehabilitation program without telecoaching	Primary outcome: 6MWT. Secondary outcomes: respiratory function; upper and lower extremity muscle strength; perception of dyspnea; quality of life; and psychological symptoms (anxiety and depression).
Elbanna et al.	2022	Egypt	2020.11-2021.03	Seniors aged 60-70 years with post-COVID-19 syndrome.	100	63.55 ±2.83	NA	NA	NA	NA	NA	Photobiomodulation treatment (PBMT), three times per week for four weeks	Placebo Photobiomodulation (PBM), three sessions per week for four weeks	Fatigability and function

SD: standard deviation; IQR: interquartile range; HGS: hand grip strength; 6MWT: six-minute walking test; FIM: functional independence measure; PCSF: Post-COVID-19 Functional Scale; STS: sit-to-stand; HADS: Hamilton Anxiety and Depression Scale; BI: Barthel Index; TUG: timed-up-and-go; STS: sit-to-stand; CAS-I: Cumulated Ambulation Score-Italian version; ICU: intensive care unit.

Table S2. Subgroup analysis of 6-minute walk test by mean age, intervention, and severity in acute infection.

Subgroups	Records, No.	Participants, No.	MD (95% CI)	p-value	Weight (%)	I^2 (%)	p-heterogeneity
Intervention							
Exercise training	2	96	19.84 (8.13, 31.56)	0.001	65.03	92.8	<0.001
Respiratory rehabilitation	3	210	14.37 (-18.72, 47.46)	0.395	28.79	70.2	0.035
Others	1	81	1.93 (-35.83, 39.69)	0.920	6.19	-	-
Severity in acute infection							
Non-severe	3	168	22.36 (10.58, 34.13)	<0.001	71.76	88.1	<0.001
Severe/critical	3	219	-2.40 (-17.60, 12.79)	0.757	28.24	0.0	0.864
Mean age							
60-65 years old	3	138	19.01 (8.05, 29.96)	0.001	70.24	85.9	0.001
>65 years old	3	249	12.83 (-19.08, 44.74)	0.431	29.76	70.4	0.034
Overall	6	387	15.77 (5.40, 26.13)	0.003	100.00	78.5	<0.001

MD: difference in mean; CI: confidence interval.

Table S3. Subgroup analysis of 30s sit-to-stand test by mean age and severity in acute infection.

Subgroups	Records, No.	Participants, No.	MD (95% CI)	P-value	Weight (%)	I^2 (%)	p-heterogeneity
Severity in acute infection							
Non-severe	4	168	5.22 (3.72, 6.72)	<0.001	70.55	85.4	<0.001
Severe/critical	2	82	1.94 (0.05, 3.82)	0.044	29.45	33.0	0.222
Mean age							
60-65 years old	2	146	5.05 (2.89, 7.22)	<0.001	57.42	94.4	<0.001
>65 years old	3	104	3.00 (1.17, 4.82)	0.001	42.58	44.6	0.165
Overall	6	250	4.11 (2.46, 5.76)	<0.001	100.00	89.9	<0.001

MD: difference in mean; CI: confidence interval.

Table S4. Subgroup analysis of hand grip strength by mean age, intervention and severity in acute infection.

Subgroups	Records, No.	Participants, No.	MD (95% CI)	p-value	Weight (%)	I^2 (%)	p-heterogeneity
Intervention							
Exercise training	5	227	1.78 (-1.36, 4.92)	0.267	72.71	90.6	<0.001
Respiratory rehabilitation	1	96	2.20 (-0.14, 4.54)	0.065	16.29	-	-
Others	1	81	0.35 (-4.56, 5.26)	0.889	11.00	-	-
Severity in acute infection							
Non-severe	3	145	1.95 (-2.89, 6.80)	0.429	48.48	88.7	<0.001
Severe/critical	4	259	1.81 (0.42, 3.19)	0.011	51.52	0.0	0.927
Mean age							
60-65 years old	2	123	-1.80 (-2.08, -1.52)	<0.001	26.03	88.7	<0.001
>65 years old	5	281	2.53 (1.29, 3.78)	<0.001	73.97	7.0	0.367
Overall	7	404	1.67 (-0.84, 4.18)	0.193	100.00	88.7	<0.001

MD: difference in mean; CI: confidence interval.

Table S5. Subgroup analysis of pulmonary function (FEV1/FVC, %) by mean age, intervention and severity in acute infection.

Subgroups	Records, No.	Participants, No.	MD (95% CI)	p-value	Weight (%)	I^2 (%)	p-heterogeneity
Intervention							
Exercise training	1	50	0.70 (-1.02, 2.42)	0.425	41.09	-	-
Respiratory rehabilitation	2	114	6.24 (3.16, 9.32)	<0.001	58.91	8.8	0.295
Severity in acute infection							
Non-severe	1	72	6.92 (4.07, 9.77)	<0.001	37.71	-	-
Severe/critical	2	92	0.79 (-0.88, 2.47)	0.353	62.29	0.0	0.631
Mean age							
60-65 years old	2	92	0.79 (-0.88, 2.47)	0.353	62.29	0.0	0.631
>65 years old	1	72	6.92 (4.07, 9.77)	<0.001	37.71	-	-
Overall	3	164	3.45 (-1.43, 8.33)	0.166	100.00	85.1	0.001

MD: difference in mean; CI: confidence interval.

Table S6. Subgroup analysis of fatigue by mean age, intervention and severity in acute infection.

Subgroups	Records, No.	Participants, No.	SMD (95% CI)	p-value	Weight (%)	I^2 (%)	p-heterogeneity
Intervention							
Exercise training	3	122	-0.88 (-1.26, -0.51)	<0.001	68.36	0.0	<0.001
Others	1	100	-0.13 (-0.52, 0.26)	0.512	31.64	-	-
Severity in acute infection							
Non-severe	3	172	-0.66 (-1.32, -0.01)	0.048	74.65	73.5	0.023
Severe/critical	1	50	-0.72 (-1.29, -0.14)	0.014	25.35	-	-
Mean age							
60-65 years old	2	150	-0.39 (-0.95, 0.18)	0.184	56.99	63.4	0.098
>65 years old	2	72	-1.01 (-1.50, -0.52)	<0.001	43.01	0.0	0.777
Overall	4	222	-0.66 (-1.13, -0.19)	0.006	100.00	62.9	0.044

SMD: standard mean difference; CI: confidence interval.

Table S7. Subgroup analysis of quality of life by mean age, intervention and severity in acute infection.

Subgroups	Records, No.	Participants, No.	MD (95% CI)	p-value	Weight (%)	I^2 (%)	p-heterogeneity
**Physical functioning**							
Intervention							
Exercise training	3	146	8.68 (2.86, 14.49)	0.003	66.82	97.0	0.000
Respiratory rehabilitation	2	114	16.50 (8.80, 24.21)	0.000	33.18	29.0	0.235
Severity in acute infection							
Non-severe	3	168	9.97 (3.52, 16.43)	0.002	78.68	98.3	<0.001
Severe/critical	2	92	16.44 (2.56, 30.33)	0.020	21.32	47.5	0.168
Mean age							
60-65 years old	4	260	8.59 (3.07, 14.10)	0.002	75.11	95.6	<0.001
>65 years old	1	72	18.30 (14.92, 21.68)	<0.001	25.89	-	-
Overall	5	260	11.41 (5.59, 17.24)	<0.001	100.00	96.8	0.000
**Bodily pain**							
Intervention							
Exercise training	3	146	5.20 (2.67, 7.73)	<0.001	71.29	85.7	0.001
Respiratory rehabilitation	2	114	15.39 (11.87, 18.92)	<0.001	28.71	0.0	0.981
Severity in acute infection							
Non-severe	3	168	7.68 (4.07, 11.29)	<0.001	90.70	95.0	<0.001
Severe/critical	2	92	16.14 (5.55, 26.73)	0.003	9.30	0.0	0.885
Mean age							
60-65 years old	4	260	5.42 (2.90, 7.95)	<0.001	75.15	80.4	0.002
>65 years old	1	72	15.40 (11.79, 19.01)	<0.001	24.85	-	-
Overall	5	260	8.48 (4.99, 11.97)	<0.001	100.00	90.9	<0.001
**General health**							
Intervention							
Exercise training	3	146	6.90 (2.83, 10.97)	0.001	69.56	93.1	0.000
Respiratory rehabilitation	2	114	8.55 (-2.32, 19.42)	0.123	30.44	68.9	0.073
Severity in acute infection							
Non-severe	3	168	7.48 (3.48, 11.48)	<0.001	81.27	94.6	<0.001
Severe/critical	2	92	9.10 (-4.79, 22.99)	0.199	18.73	72.2	0.058
Mean age							
60-65 years old	4	260	6.42 (2.57, 10.27)	0.001	76.71	89.8	<0.001
>65 years old	1	72	12.80 (9.21, 16.39)	<0.001	23.29	-	-
Overall	5	260	7.98 (4.29, 11.67)	<0.001	100.00	90.5	<0.001
**Role-physical**							
Intervention							
Exercise training	3	146	4.40 (2.20, 6.60)	<0.001	71.82	81.4	0.005
Respiratory rehabilitation	2	114	13.99 (10.65, 17.33)	<0.001	28.18	0.0	0.948
Severity in acute infection							
Non-severe	3	168	7.05 (3.48, 10.61)	<0.001	95.59	95.0	<0.001
Severe/critical	2	92	9.38 (-6.33, 25.08)	0.242	4.41	0.0	0.708
Mean age							
60-65 years old	4	260	4.46 (2.33, 6.59)	<0.001	73.55	73.2	0.011
>65 years old	1	72	14.00 (10.63, 17.37)	<0.001	26.45	-	-
Overall	5	260	7.13 (3.72, 10.54)	<0.001	100.00	90.2	<0.001

**Vitality**							
Intervention							
Exercise training	3	146	6.17 (3.07, 9.27)	<0.001	67.61	77.8	0.011
Respiratory rehabilitation	2	114	13.16 (7.78, 18.53)	<0.001	32.39	20.8	0.261
Severity in acute infection							
Non-severe	3	168	8.77 (4.16, 13.38)	<0.001	82.56	93.7	<0.001
Severe/critical	2	92	5.48 (-2.49, 13.45)	0.178	17.44	0.0	0.835
Mean age							
60-65 years old	4	260	6.20 (3.37, 9.03)	<0.001	74.72	66.8	0.029
>65 years old	1	72	14.30 (11.13, 17.47)	<0.001	25.28	-	-
Overall	5	260	8.19 (4.20, 12.18)	<0.001	100.00	87.4	<0.001
**Social functioning**							
Intervention							
Exercise training	3	146	4.74 (1.67, 7.82)	0.002	69.62	85.7	0.001
Respiratory rehabilitation	2	114	14.00 (3.80, 24.20)	0.007	30.38	46.3	0.172
Severity in acute infection							
Non-severe	3	168	6.57 (3.03, 10.12)	<0.001	91.68	93.1	<0.001
Severe/critical	2	92	13.09 (-5.64, 31.82)	0.171	8.32	62.9	0.101
Mean age							
60-65 years old	4	260	5.41 (2.03, 8.78)	0.002	73.40	83.8	<0.001
>65 years old	1	72	11.00 (7.85, 14.15)	<0.001	26.60	-	-
Overall	5	260	7.09 (3.59, 10.59)	<0.001	100.00	88.0	<0.001
**Mental health**							
Intervention							
Exercise training	3	146	4.56 (2.56, 6.57)	<0.001	69.26	74.6	0.019
Respiratory rehabilitation	2	114	6.15 (-5.55, 17.84)	0.303	30.74	88.7	0.003
Severity in acute infection							
Non-severe	3	168	6.46 (3.28, 9.65)	<0.001	78.51	92.1	<0.001
Severe/critical	2	92	2.65 (-2.52, 7.83)	0.315	21.49	16.2	0.275
Mean age							
60-65 years old	4	260	4.24 (2.26, 6.23)	<0.001	79.17	68.4	0.023
>65 years old	1	72	11.70 (8.35, 15.06)	<0.001	20.83	-	-
Overall	5	260	5.61 (2.88, 8.34)	<0.001	100.00	85.3	<0.001
**Role-emotional**							
Intervention							
Exercise training	3	146	3.86 (3.04, 4.67)	<0.001	69.18	0.0	0.504
Respiratory rehabilitation	2	114	14.76 (11.50, 18.02)	<0.001	30.82	0.0	0.534
Severity in acute infection							
Non-severe	3	168	7.16 (2.55, 11.76)	0.002	92.12	95.1	<0.001
Severe/critical	2	92	11.85 (-2.64, 26.34)	0.109	7.88	0.0	0.635
Mean age							
60-65 years old	4	260	3.86 (3.04, 4.68)	<0.001	72.10	0.0	0.696
>65 years old	1	72	14.90 (11.61, 18.19)	<0.001	27.90	-	-
Overall	5	260	7.51 (3.15, 11.86)	0.001	100.00	90.5	<0.001

MD: difference in mean; CI: confidence interval.

Table S8. Subgroup analysis of anxiety by mean age, intervention and severity in acute infection.

Subgroups	Records, No.	Participants, No.	SMD (95% CI)	p-value	Weight (%)	I^2 (%)	p-heterogeneity
Intervention							
Exercise training	2	96	-1.27 (-1.71, -0.83)	<0.001	49.14	0.0	0.548
Respiratory rehabilitation	2	114	-0.36 (-1.81, 1.09)	0.628	50.86	92.6	0.000
Severity in acute infection							
Non-severe	3	168	-1.19 (-1.52, -0.86)	<0.001	75.27	0.0	0.723
Severe/critical	1	42	0.39 (-0.22, 1.00)	0.207	24.73	-	-
Mean age							
60-65 years old	3	138	-0.72 (-1.82, 0.39)	0.203	73.87	89.5	<0.001
>65 years old	1	72	-1.09 (-1.58, -0.59)	<0.001	26.13	-	-
Overall	4	210	-0.81 (-1.58, -0.05)	0.038	100.00	85.4	<0.001

SMD: standard mean difference; CI: confidence interval.

Table S9. Subgroup analysis of depression by mean age, intervention and severity in acute infection.

Subgroups	Records, No.	Participants, No.	SMD (95 %CI)	p-value	Weight (%)	I^2 (%)	p-heterogeneity
Intervention							
Exercise training	2	96	-1.67 (-2.14, -1.20)	<0.001	48.75	0.0	0.365
Respiratory rehabilitation	2	114	-0.16 (-0.53, 0.21)	0.399	51.25	0.0	0.518
Severity in acute infection							
Non-severe	3	168	-1.18 (-2.23, -0.14)	0.026	75.03	89.3	<0.001
Severe/critical	1	42	0.00 (-0.61, 0.61)	1.000	24.97	-	-
Mean age							
60-65 years old	3	138	-1.12 (-2.26, 0.03)	0.057	73.72	89.5	<0.001
>65 years old	1	72	-0.25 (-0.72, 0.21)	0.288	26.28	-	-
Overall	4	210	-0.89 (-1.76, -0.02)	0.046	100.00	88.5	<0.001

SMD: standard mean difference; CI: confidence interval.

Table S10. Publication bias.

**Outcome variables**	Root MSE	p-value[a]
**Functional capacity**		
6-minute walk test, m	2.413	0.987
30 s sit-to-stand test, n	2.751	0.186
Hand grip strength, kg	1.619	0.011
**Fatigue**	0.204	0.005
**Pulmonary Function**		
FEV1/FVC, %	3.281	0.707
**Quality of life**[b]		
Physical functioning	5.098	0.272
Bodily pain	3.278	0.375
General health	3.628	0.687
Role-physical	3.284	0.445
Vitality	3.122	0.649
Social functioning	2.800	0.347
Mental health	2.881	0.641
Role-emotional	3.185	0.362
**Independence in activities of daily living**	0.943	0.638
**Depression and Anxiety**		
Depression	2.497	0.279
Anxiety	3.157	0.821

MSE: mean square error; FEV1/FVC: forced expiratory volume in one second/forced vital capacity. [a] P<0.1 was considered evidence of publication bias.

Table S11. Sensitivity analysis by trimming and filling method.

Outcome variable	Before trimming and filling		After trimming and filling	
	MD (95% CI)	p-value	MD (95% CI)	p-value
Hand grip strength, kg	1.67 (-0.84, 4.18)	0.193	0.22 (0.03, 1.77)	0.156
Fatigue	-0.66 (-1.13, -0.19)[a]	0.006	-0.66 (-1.13, -0.19)[a]	0.006

MD: difference in mean; CI: confidence interval. [a] Using standard mean difference (SMD) as pooled effect size.
